# Effects of Al(III) and Nano-Al_13_ Species on Malate Dehydrogenase Activity

**DOI:** 10.3390/s110605740

**Published:** 2011-05-27

**Authors:** Xiaodi Yang, Ling Cai, Yu Peng, Huihui Li, Rong Fu Chen, Ren Fang Shen

**Affiliations:** 1 Jiangsu Key Laboratory of Biofunctional Materials, Laboratory of Electrochemistry, College of Chemistry and Material Science, Nanjing Normal University, Nanjing 210097, China; E-Mails: yangxiaodi@njnu.edu.cn (X.Y.); linglingcai@163.com (L.C.); 542053746@qq.com (H.L.); 2 State Key Laboratory of Soil and Sustainable Agriculture, Institute of Soil Science, Chinese Academy of Sciences, Nanjing 210008, China; E-Mail: rfchen@issas.ac.cn (R.F.S.)

**Keywords:** electroanalysis, spectroscopy, Al(III), Al_13_, malate dehydrogenase

## Abstract

The effects of different aluminum species on malate dehydrogenase (MDH) activity were investigated by monitoring amperometric *i*-t curves for the oxidation of NADH at low overpotential using a functionalized multi-wall nanotube (MWNT) modified glass carbon electrode (GCE). The results showed that Al(III) and Al_13_ can activate the enzymatic activity of MDH, and the activation reaches maximum levels as the Al(III) and Al_13_ concentration increase. Our study also found that the effects of Al(III) and Al_13_ on the activity of MDH depended on the pH value and aluminum speciation. Electrochemical and circular dichroism spectra methods were applied to study the effects of nano-sized aluminum compounds on biomolecules.

## 1. Introduction

Aluminum is the most abundant element in the Earth’s crust, and since 1980s a considerable release of aluminum to the environment has occurred due to the increased input of acids, which has brought about a burgeoning human exposure to aluminum during everyday life activities and poisoning of the ecological system as well as damage to human health [1–3]. It has been reported that the major constituent of antiperspirants are aluminum salts which have long been associated with cancer [4] as well as other human diseases [5]. Aluminum is an important relevant etiological factor in several diseases, such as dialysis dementia, Alzheimer’s disease, Parkinson’s disease, osteomalacia and anemia [6–10]. It is a pro-oxidant and can significantly increase the potential for oxidative damage in the skin [11,12]. Recent *in vitro* studies have shown that Al(III) could inhibit the activity of enzymes which catalyze the tricarboxylic acid (TCA) and glycolytic cycles, thereby the production of energy generated by mitochondrial cell was negatively affected [13]. It has been suspected that the toxicity of aluminum depends on its various polynuclear species that exist at different pH values. Therefore, it is reasonable to presume that different aluminum species may display different effects on the activity of biological enzymes.

In recent years, the widespread use of novel nanoparticles has led to unforeseen health or environmental hazards to humans or other species, and the diversity of health and safety regulations has puzzled us [14]. With thousands of novel materials under investigation, there are several reports on the toxicity of nanomaterials, such as fullerene and its derivatives, quantum dots, nano-oxides (titanium dioxide, silica, zinc oxide, alumina, *etc.*) [15–19]. As a new type clarifying agent, nanometer-sized tridecameric aluminum polycation (nano-Al_13_, also called nano-polynuclear Al_13_) shows more effective coagulation effects and rapid aggregation rates over a relatively wider pH range, and is widely applied in water treatment. It is also used in a number of commercial products such as pillared agents, antacids, antiperspirants and surface active agents [20–23]. This has given rise to a debate as to whether Al_13_ exists ubiquitously in Nature, but there are some reports about Al_13_ formation in the environment. For example, it has been found that Al_13_ could be formed under some natural conditions such as in streams and lakes where water of differing acidity is mixed and in groundwater areas where water flows through basic materials [24]. Hunter *et al.* found Al_13_ in organic soils in acid forests in America [25], but Masion *et al.* called into question that report [26]. It has been reported that nano-Al_13_ is probably the real species under the physiological condition, and the Al(OH)_3_ formation process requires the presence of nanopolynuclear Al_13_ as a precursor. Furrer *et al.* found that in some acidic waters, flocculent deposits of Al were formed by the accumulation of Al_13_ [27]. V. Rao and J. Rao [28] have provided evidence for the presence of the Al_13_ polymer inside the synaptosomes, but this has aroused controversy among researchers. It has been found that Al_13_ has 10-fold higher toxicity to plant roots compared to monomeric Al^3+^ and toxicities comparable to monomeric Al in algae [29]. Al_13_ introduced into lakes and rivers may be toxic to fish [30]. In this paper, we have studied the biological effects of Al_13_ *in vitro* under physiological conditions. Malate dehydrogemase (MDH) exists in all creatures and has been isolated from many diverse sources [31]. It is a key enzyme in eukaryotic and prokaryotic cells that catalyzes the reversible conversion between malate and oxaloacetate with strict substrate specificity in the presence of the coenzyme nicotinamide adenine dinucleotide [NAD(H)]. The biological functions of MDH are various, including energy generation in mitochondria, reactive oxygen species metabolism in plants, *etc.* It is also essential to many metabolic pathways such as the TCA cycle, photosynthesis, C4 cycle and so on. Porcine heart MDH has been extensively used as a model oligomeric enzyme for structural and kinetic studies to explain the significance for catalysis or allosteric regulation [32,33].

In our previous investigation, we examined the effects of Al(III) and nano-Al_13_ species on the activity of glutamate dehydrogenase (GLDH) by electrochemical methods using a functionalized MWNT-GCE. It is weel known that electrochemical analysis is a powerful tool to trace metal ions and biomolecules in biological systems with a number of remarkable advantages such as high sensitivity, faster and more reliable results, simple instrumentation and operation procedures and lower cost. In the past few decades, the analysis of enzymes by electrochemical methods has been reported. Gao and Xin [34] studied the effects of lanthanide ions on the kinetics of GLDH by a chronoamperometric method using a bare glassy carbon electrode (GCE). Bi *et al.* investigated the effect of Al(III) and Al_13_ on the activity of LDH by differential pulse voltammetry using a hanging mercury drop electrode (HMDE) [35]. Yang *et al.* have reported their electrochemical studies on the inhibition and activation effects of Al(III) on the activity of bovine liver GLDH with HMDE [36]. In recent years, more and more researchers have turned their attention to the application of modified electrodes in biochemistry for their excellent catalytic properties. Zhuang *et al.* [37] have explored the electrochemical properties of unfunctionalized single-walled carbon nanotubes (SWNT) as nanometer-sized activators in enzyme-catalyzed reactions and experiments showed that the modified electrodes could be successfully used to monitor the activity of LDH.

The effects of a number of regulators, such as anions [38], metal cations [39], amino acids and nucleotides [40] on the activity of MDH from various organisms have been studied. However, so far there has been no study on the effects of Al species compounds on the activity of the enzyme MDH. In this work, an electrochemical technique was used to detect the oxidation current of NADH at low potential which can describe the MDH activity. Based on the sensitive and stable *i*-t curve of NADH on the modified electrode, we successfully explored the effects of various aluminum species, especially nano-Al_13_, on the activity of MDH. Meanwhile, some other factors which may influence MDH activities were also investigated.

## 2. Materials and Methods

### 2.1. Materials and Instrumentation

Malate dehydrogenase (MDH, EC, 1.1.1.37) from porcine heart, oxaloacetate and β-nicotinamide adenine dinucleotide (NADH, NAD^+^) were purchased from Sigma (St. Louis. MO, USA). Multi-walled carbon nanotube (MWNT, less than 10 nm in diameter and 0.5–500 nm in length with purity > 95%) was obtained from Shenzhen Nanotech Port Co., Ltd. (Shenzhen, China). All other chemicals were of analytical reagent grade. All solutions were prepared with doubly distilled water. Tris-HCl buffer solution was prepared by dissolving an appropriate amount of tris(hydroxymethyl)-aminomethane and then adjusting the pH value with concentrated HCl. The Al stock solution was prepared by dissolving high purity metallic Al powder (99.99%) in hydrochloric acid at pH < 2 to prevent hydrolysis of Al^3+^ ion. Then the stock solution was diluted with doubly distilled water. Electrochemical measurements were performed using a CHI660B electrochemical system (CH Instruments, Chenhua Inc., Shanghai, China). A three-electrode configuration, which was employed, consist of a MWNT-modified GC or a bare GC electrode as a working electrode, platinum wire and saturated calomel electrode (SCE) as auxiliary electrode and reference electrode, respectively. Before the electrochemical experiments, the Tris-HCl buffer solution was degassed for at least 20 min by bubbling high-purity nitrogen gas and the solution was kept in a nitrogen environment to prevent oxidation. Circular dichroism (CD) spectra were measured on a JASCO-810 instrument.

### 2.2. Preparation of MWNT-CHIT Modified Electrode

Twenty milligrams of MWNT were dispersed in 30% HNO_3_ (30 mL) and the resulting mixture was then refluxed for 24 h at 140 °C as described in reference [41]. The resulting suspension was centrifuged and the precipitate was washed with water to obtain carboxylic group functionalized MWNT. The obtained functionalized MWNT was completely dispersed in water at pH 1.0 to prepare a 5 mg mL^−1^ MWNT suspension, which was then neutralized to pH 7.0 with 0.1 M NaOH. A 1.0 wt% chitosan (CHIT) stock solution was prepared by dissolving chitosan flakes in hot (80–90 °C) aqueous solution of 1% HOAc. The solution was cooled to room temperature, and its pH was adjusted to 3.5–5.0 using a concentrated NaOH solution. A MWNT- CHIT solution was prepared by mixing the obtained functionalized MWNT suspension and the CHIT solution. The aqueous mixture was then sonicated for 30 min to obtain a homogeneous suspension. The glassy carbon electrode (GCE) was successively polished to a mirror finish using 0.3-μm and 0.5-μm alumina slurry followed by rinsing thoroughly with doubly distilled water. After successive sonication in absolute alcohol and doubly distilled water, the electrode was rinsed with doubly distilled water and allowed to dry at room temperature. The MWNT-CHIT modified electrode was prepared by casting 1.2 μL of MWNT-CHIT solution on the surface of GCE, and then was dried at room temperature. The electrode was stored in a desiccator when not in use.

### 2.3. Synthesis of Nano-Polynuclear Aluminum Sulfate

A 0.25 M aluminum chloride solution (25 mL) was heated and kept a 70 °C using a thermostat. Then 0.25 M NaOH solution (60 mL) was slowly added with accurate control of the rate of addition under continuous stirring. The obtained solution was kept 24 h at room temperature, then 0.1 M Na_2_SO_4_ solution (62.5 mL) was added. After ten min. of reaction, the solution was filtered and then aged for 48 h. The crystals isolated by centrifugation were washed twice with distilled water and 70% ethanol solution, respectively, then air-dried and stored in a desiccator for future use. The X-ray diffraction (XRD) and NMR spectra of the obtained crystal matched well with previous reports [42,43], which proved that the nano-polynuclear aluminum sulfate has been successfully synthesized by our proposed method.

### 2.4. Effects of Aluminum Species and Interference Factors on MDH Activity in the MDH Reaction System

Tris-HCl butter solution (5 mL) was transferred into the electrolytic cell, and degassed for 10 min by bubbling N_2_ gas through it. The experiment was carried out at a constant temperature and a N_2_ atmosphere was kept over the solution throughout the measurement. A measured amount of different Al species stock solution or other factors such as GSSG and d-Glu were transferred into the electrolytic cell and the solution was stirred magnetically. Then 0.01 mM oxaloacetate (20 μL) and 0.01 mM NADH (20 μL) were added into the electrolytic cell. After injecting MDH into the assay, the enzymatic reaction initiated and the oxidation current of NADH were recorded in amperometric *i*-t curve mode on the MWCNT-modified electrode.

### 2.5. Determination of Michaelis Constant K_m_ and Maximum Velocity V_max_ for NADH

Five milliliters of Tris-HCl buffer solution was added into the electrolytic cell and degassed for 10 min by bubbling nitrogen gas through it. The experiment was carried out at a constant temperature and a nitrogen atmosphere was kept over the solution throughout the measurement. Then 0.01 mM oxaloacetate (20 μL) and 0.1 M NADH (10, 20, 30, 40, 50 μL) were added to the electrolytic cell. The oxidation currents of NADH were recorded in amperomentic *i*-t mode. The Michaelis Constant *K_m_* and Maximum Velocity *V_max_* for NADH were calculated.

## 3. Results and Discussion

### 3.1. Electrochemical Response of MWNT-CHIT Modified Electrode to NADH

**Figure 1.**
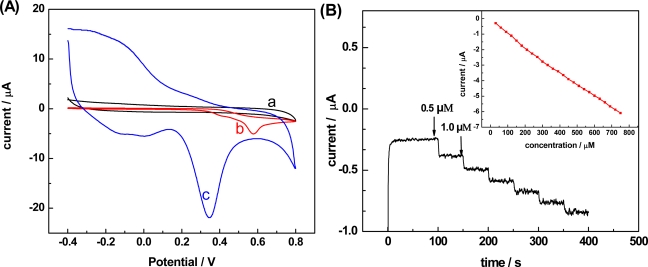
(**A**) Cyclic voltammogrames of the oxidation of NADH on (**a**) bare electrode without NADH; (**b**) bare electrode in 5 mL pH 7.5 Tris-HCl buffer solution containing 0.5 mM NADH; (**c**) MWNT-modified electrode in 5 mL pH 7.5 Tris-HCl buffer solution containing 0.5 mM NADH. Scan rate, 10 mV s^−1^. (**B**) Amperometric responses of the MWNT-modified electrode to NADH in Tris-HCl buffer solution (pH 7.5) at 0.32 V. The addition of NADH is 5 μL each time at the interval of 50 s.

Figure 1(A) shows the cyclic voltammograms of the bare and MWNT-CHIT modified electrodes in 5 mL tris-HCl buffer solution (pH 7.5) containing 0.5 M NADH recorded with a scan rate of 10 mV s^−1^. No peak was observed with a bare GCE (curve a) without adding NADH. Upon the addition of NADH, the oxidation resulted in an oxidized peak with the anodic potential of +0.6 V *vs.* SCE at the bare GCE (curve b), whereas the modified electrode exhibited a dramatic enhancement in the peak value of NADH oxidation at 0.32 V (curve c).

The pair of broad redox peaks appearing in curve c at the potentials of −0.16 V and −0.10 V could be ascribed to the reduction and oxidation of oxygen-containing groups on the functionalized MWNT surface, respectively, in agreement with a previous report [41]. The background currents of curve c are much larger than those of curves a and b, which may be attributed to the fact that the apparent surface area of MWNT-GCE is larger than that of a bare GCE. The substantial negative shift of the anodic peak demonstrated that the modified electrode is very effective in promoting the electrochemical oxidation of NADH. The stability of the modified electrode was studied by repetitively cycling the electrode in a solution of NADH, and it was found that the modified electrode could retain 96% of its initial current response after 50 repetitive scanning cycles. The storage stability of modified electrode was examined by checking the response current periodically and it only showed 8% loss of initial value after a storage period of three weeks. The results suggested that the proposed modified electrode could successfully decrease the overpotential of the oxidation of NADH, and displayed perfect stability for the oxidation of NADH.

The analytical performance of the modified electrode was tested by recording the amperometric response of the oxidation of NADH. Figure 1(B) shows the amperometric *i*-t curve for the oxidation of NADH on the MWNT-CHIT modified electrode with a fixed potential of 0.32 V in a stirred Tris-HCl of pH 7.5 and NADH was injected at regular interval. A gradual increase in the current with each addition was observed and the amperometric response was stable upon repeating the injection. The response displayed a linear range from 0.3 to 750 μM with a correlation coefficient of 0.999 and slope of 7.9 nA/μM. The electrocatalytic behavior was highly reproducible, as reflected by relative standard deviations of 3.9 and 4.2% for five determinations at NADH concentrations of 3.0 and 150 μM. A relative standard deviation of 3.2% was estimated from the slope of the calibration plots at five freshly prepared MWNT-modified electrodes. The excellent analytical performance of the modified electrode indicates that it can be used to detect the electrochemical signal change of NADH in the catalytically reaction of oxaloacetate by MDH which was used to describe the activity of MDH. It has been reported that at the beginning of the enzyme-catalyzed reaction, the current *I_t_* decreased linearly with time in a short time span. According to that, the initial rate (v_0_) could be calculated from the initial slope of the enzyme-catalytic reaction progress curve which was used to describe the enzymatic activity.

The Fourier Transform infrared (ATR-FTIR) spectrum could further confirm the fine immobilization of MWNTs. The oxygen-containing groups on the MWNTs introduced during the purified process as a medium of electrons in the process of the oxidation of NADH made the electron transferring easier, so the successfully introduced oxygen-containing groups could effectively enhance the oxidation of NADH.

### 3.2. The Electrochemical Response of Enzyme-Catalyzed Reaction

Malate is a key product of plant metabolism and thought to be the ultimate product of glycolysis [44]. The enzyme-catalyzed reaction of oxaloacetate reduction by MDH utilizing NADH as coenzyme can be expressed as follows:

$$\text{Oxaloacetate}+\text{NADH}+{\text{H}}^{+}↔\text{Malate}+{\text{NAD}}^{+}$$

**Figure 2.**
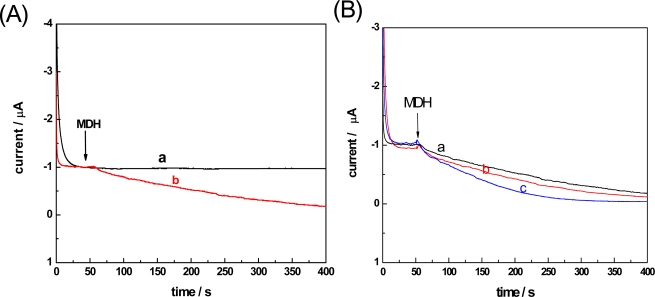
Amperometric response of the MWNT-modified electrode to the oxidation of NADH in a well-stirred assay mixture: 1 mM oxaloacetate + 1 mM NADH + 100 mM Tris-HCl buffer (pH 7.5) + MDH (**A**) (**a**) 0 μL MDH (**b**) 10 μL MDH. (**B**) (**a**) no Al (III) and Al_13_ (**b**) 30 μM Al (III) (**c**) 30 μM Al_13_.

The cyclic voltammogram recorded at the modified electrode in 1M oxaloacetate +1 M NADH + 100 M Tris-HCl buffer (pH 7.5) showed that the peak potential of NADH oxidation was 0.32 V. Thus, in our amperometric *i*-t experiments, a potential of 0.32 V was set and the amperometric *i*-t curve was recorded, as shown in Figure 2(A). It is clear that there is no obvious current change without adding MDH [Figure 2(A)-a], whereas with the addition of 10 μL MDH (10 mg mL^−1^) to the assay solution, the anodic oxidative current of NADH gradually decreased [Figure 2(A)-b], which means that the MDH catalytic reaction started and finally reached reversible equilibrium. It is, therefore, evident that the amperometric *i*-t technique using the MWNT-CHIT modified electrode could accurately determine the MDH activity in an enzymatic catalytic reaction by tracing the decreasing NADH oxidation current.

### 3.3. Effects of Al(III) and Nano-Al_13_ on MDH Activity

In order to investigate the effects of Al(III) and nano-Al_13_ on the enzyme activity, 30 mM Al(III) and nano-Al_13_ was added to the assay mixture, and the amperometric *i*-t curves of the oxidation current of NADH were measured. As shown in Figure 2(B), curves b and c became sharper compared with curve a, which indicated that the consumption of NADH became faster. The results may be explained by assuming that Al(III) and nano-Al_13_ had an activating effect on the activity of the MDH enzyme and could accelerate the enzyme-catalyzed reaction. Moreover, a much larger change can be observed in curve c than in curve b, which demonstrated that the effect of nano-Al_13_ on MDH was higher than that of Al(III) species in aqueous solution. The results were further proved by the respective Michaelis constant *K_m_* and Maximum velocity *V_max_* values, as shown in Table 1.

### 3.4. Effects of Al(III) and Al_13_ on MDH Activity at Different pH Values

**Figure 3.**
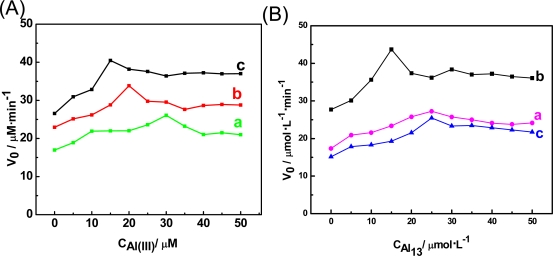
The effects of Al(III) and Al_13_ on the v_0_ of the oxaloacetate reduction reaction in a well-stirred assay mixture: 1 mM oxaloacetate + 1 mM NADH + 100 mM Tris-HCl buffer at different pH. Reaction was initiated by addition of 10 μL MDH. (**A**): (**a**) pH 8.5 (**b**) pH7.5 (**c**) pH 6.5 (**B**): (**a**) pH 8.5 (**b**) pH 7.5 (**c**) pH 6.5.

The effects of Al(III) and Al_13_ on the v_0_ of the oxaloacetate reduction reaction catalyzed by MDH at different pH values were examined successfully by the amperometric *i*-t curves. As shown in Figure 3(A,B), the activation effects of Al(III) and Al_13_ reach a maximum level with the increase of Al(III) and Al_13_ concentration, while the maximum points were different at various pH values. The possible reason is that Al(III) and Al_13_ may exist in various speciations at different pH values, and they display different biological effects on MDH. As shown in Figure 3(A), at pH 6.5 Al(III) displayed the strongest effect on MDH activity, and the effect was reduced as the pH increased. The differences of the Al(III) effect with pH may be due to fact that the Al(III) speciation gives forms that interact more effectively with the MDH enzyme and become dominant at lower pH. It is worth noticing that unlike those of Al(III), the activating effects of Al_13_ were highest at pH 7.5 [Figure 3(B)]. The phenomenon could be explained that the fact the stability of Al_13_ varied with pH. While Al_13_ is stable at pH 7.5, it may be depolymerized at pH 8.5 and 6.5.

### 3.5. Effects of Other Aluminum Species and Interference Factors on the MDH Activities

**Figure 4.**
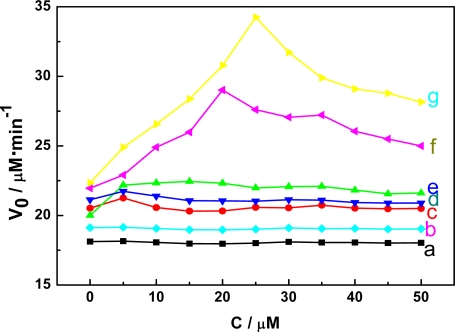
The effects of Al species and other interference factors on the activity of MDH. (pH 7.5) (**a**) GSSG (**b**) D-Glu (**c**) Al-F (**d**) Al-citrate (**e**) Al-oxalic acid (**f**) Al(III) (**g**) Al_13_.

It has been proved that not all chemical forms of aluminum are equally toxic. In our research, the effects of different species of aluminum on the MDH activity were explored as shown in Figure 4. The curves c and d indicate that Al-F and Al-citrate have little effect on the activity of MDH, and Al-oxalic acid has a small effect, as shown in curve e, which may due to their different complexation ability. Al(III) and Al_13_ influenced the MDH activity seriously and Al_13_ displayed the largest inhibition (curves f and g) and the proposed explanation is as follows: the F, citrate and oxclic acid ligands have stronger complexation ability towards Al than NADH and MDH, thereby, Al interacted preferentially with those ligands and displayed little effects on the activity of NADH and MDH. However, Al(III) and Al_13_ could interact with NADH and MDH because of the absence of other ligands in the assay. Moreover, the effects of other factors such as GSSG and D-Glu on the enzyme was also investigated, and the results showed that there is almost no effects on the activity of MDH, as shown in curves a and b.

### 3.6. Proposal Explanation of the Activating Effects of Al(III) and Al_13_ on MDH

It is known that Al speciation generally inactivates enzyme activity as shown in previous investigations [35,36], Recent research [45–47] on the chemical structure of Al complexes has shown that the Al atom binds to the oxygen atoms in carboxyl groups by coordinated bonds, but in this work we have found that some Al species could activate MDH, so an explanation is called for. The activating effect of Al(III) and Al_13_ may be explained from two aspects.

On the one hand, from the point of view of the mechanism of the enzyme-catalyzed reaction, the initial enzyme-catalyzed reactions steps involve the binding of the substrate and coenzyme to the enzyme surface and the enzyme will orient these reactants relative to each other to form an enzyme-substrate-coenzyme complex. During the reaction of oxaloacetate reduction, the NADH was oxidized to NAD^+^. After the catalyzed reaction is completed, the enzyme opens up again to let the products leave and to prepare for the next substrate, and in the whole reaction the dissociation step of enzyme-product complex was the key stage [39]. It has been proved that Al(III) and Al_13_ could strongly bind with the adenine N_7_ and pyrophosphate free oxygen function groups of NAD+ [48], so we conjectured that Al(III) and Al_13_ would interrupt the binding of NAD+ to MDH by combining preferentially to NAD^+^, which could enhance the dissociation of the NAD^+^-MDH complex. The enhanced dissociation could accelerate the equilibrium reaction of oxaloacetate reduction moving towards the negative direction.

**Figure 5.**
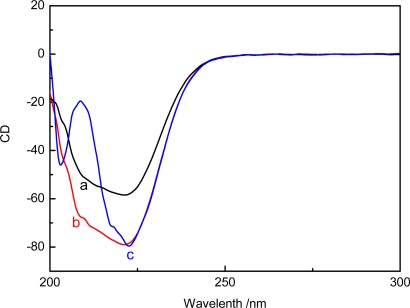
CD spectra of MDH in absence and presence of Al(III) and Al_13_ (**a**) native MDH (**b**) MDH + Al(III) (**c**) MDH+Al_13_.

On the other hand, the conformational change of MDH caused by the direct interaction of Al(III) and Al_13_ to MDH would contribute to the activating effect. To further explore the conformational change of MDH, circular dichroism (CD) was used to indicate that the binding of aluminum to the enzyme would induce a transformation between α-helices and β-sheets and random coils, which results in a change of enzyme activity. The CD spectral of MDH undergoes a rapid change in the presence of Al(III) and Al_13_, as shown in Figure 5. We can observe a increase in the CD spectra intensity of MDH in the far-UV region upon addition of Al(III) (curve b), which indicates an alteration in the secondary structure of the enzyme. It is well known that the split-hump in the far-UV region is the typical spectrogram of the α-helix structure of proteins [49], so the appearance of a split-hump in the far-UV region in the CD spectra of MDH upon addition of Al_13_ indicates that the presence of Al_13_ could increase the amount of α-helix structure of MDH, which may make it more easy for the substrate to access the active site of the enzyme.

The reason that Al_13_ exhibited a stronger effect on the activity of MDH may be on account of the larger surface effects of Al_13_ clusters formed in aqueous solution. A model has been built to show the backbone structure of the aluminum clusters, from which we may draw the conclusion that Al(III) clusters could exert a strong effect on biomolecules. The increased active sites on the surface make it possible to develop more significant interactions between ligands and the cluster surface. Of course, there are 13 aluminum atoms in one Al_13_ molecule which is 13-fold more than in than free Al(III) and this automatically results in a greater effect for the concentration. However, we viewed Al_13_ as an independent species to discuss the inhibitory ability rather than as 13 aluminum atoms. As a matter of fact, the interaction between Al_13_ and biomolecules was not simply a “sample plus 13 free Al(III) atoms” one. In our studies, we paid attention to investigating the efficacy of aluminum speciation on the biological system. Of course, our proposal explanation could primarily discuss the activating effects of Al species on the activity of MDH. However, it is indeed true that aqueous Al would have undergone a process of ongoing Al polymerization, and the toxicity of aluminum is a matter of reaction kinetics, where the degree of ongoing Al polymerization is the most important [50], so it is both significant and valuable to explore more thoroughly the mechanism.

## 4. Conclusions

In summary, the effects of aluminum species on the activity of MDH have been successfully investigated by electrochemical methods on a functionalized MWNT-GCE. The results showed that the proposed technique was a powerful tool for the determination of the enzyme activity. Our studies also showed that the effects of different aluminum species on the activity of MDH were not equal, and the nanopolynuclear Al_13_ displayed the strongest effect on the enzyme activity. At the meantime, the proposed mechanism of the effect of aluminum was discussed from several different points of view. However, the interaction between aluminum and the enzyme system was complicated by the mechanisms of the molecular interactions and the surface action needs to be further studied.

## Figures and Tables

**Table 1.** The effects of Al(III) and Al_13_ on *K_m_* and *V_max_* of MDH (pH 7.5, 25 °C).

	
	***Vmax* (mM·min^−1^)**	***Km* (μM)**
Al(III) (μM)		
0	28.1	23.8
30	30.3	16.1
Nano-Al_13_ (μM)		
0	28.3	23.5
30	28.5	13.6
